# A Method for Making Seamless Gold Crowns

**Published:** 1899-03

**Authors:** Mark Hayter

**Affiliations:** Dallas, Ore.


### A METHOD FOR MAKING SEAMLESS GOLD CROWNS.[1]

BY DR. MARK HAYTER, DALLAS, ORE.

Mr. President, and Members of the Oregon State Dental Association,—I will attempt to describe to you a method I have devised for making seamless gold crowns. The tooth or root to be crowned is prepared in the usual manner, and measured with a thin strip of copper or a fine wire. Select a draw-plate punch that will just pass through the measure and lay to one side for future use; cut and straighten the measure; from a piece of well-annealed copper plate (the same gauge as the gold used in the draw-plate) cut a strip the length of the measure and about three-fourths the width the finished crown is to be in length. Bend this strip, bringing the ends together, and solder; this can be done with silver solder over an alcohol lamp or gas-jet. Shape and fit this band to the tooth, trimming the edge so that it will pass under the free margin of the gum; also trim the other edge, if necessary, to prevent the opposing teeth from interfering when in occlusion. Remove the band, and with suitable pliers give the desired contour. Use vaseline in oiling the tooth on which the crown is to be placed, as also the opposing teeth, and place the band in position, wedging if necessary to keep in place. Fill with quick-setting plaster, and have the patient close the jaws and keep them so until the plaster sets. This gives the correct occlusion. Remove the band and trim the plaster to represent the remaining portion of the crown. This band and the plaster combined give the outward form of the finished crown. I call this the "crown form." Finish filling the band with plaster or moldine, and into this insert a burnt match, wood toothpick, or anything by which it can be most conveniently handled. Pour a rubber ring about an inch deep nearly full of fusible metal, and into this press the "crown form" full length, occlusal end down. Remove the plaster, and with a medium-sized wheel bur make a groove in the metal around the upper edge of the band. Pinch the band together and remove. The mould for the crown is now complete. Draw a cartridge of gold to the size of the punch previously selected and shape it so that it will enter the mould. Into this, with suitable punches, drive shot and cotton until it is swaged to the form of the mould. A little oil dropped

[1] Read before the Oregon State Dental Association, December 15, 1898.

into the shot before the swaging is commenced will prevent them from being driven into a solid mass, and makes their removal an easy matter. Place the mould in a ladle and melt away from the crown. Trim the neck of the crown to the line made by the groove in the mould, polish, and, if the work has been done carefully, it is ready to be cemented to place.

When crowning a tooth to be used as an abutment in bridge-work, if but little if any of the occlusal surface is missing, the method differs from the above in the following respect: After shaping the tooth and fitting the band, with modelling compound take the bite, and, if the band does not come with it, remove, place in proper position, pour each side, and place in a crown articulator. After the plaster has set remove the compound and varnish the opposing teeth, place a little fresh-mixed plaster upon the end of the tooth within the band, close the articulator, and allow it to remain so about five minutes. Open and trim the plaster to the desired shape; then cut the "crown form" from the cast and proceed to finish as first described.

As you may have noticed, this method is made up in the main from parts of other methods. About the only original feature claimed is the sinking of the "crown form" into the fusible metal, full length, thereby making a mould or counter-die in which the crown is formed.

The merits claimed for crowns made by this system are perfect occlusion, proper contour, accurate adjustment, and the ease and rapidity with which the work can be done.

